# Characterization of Platelet Receptors and Their Involvement in Immune Activation of These Cells

**DOI:** 10.3390/ijms252312611

**Published:** 2024-11-24

**Authors:** Beata Tokarz-Deptuła, Łukasz Baraniecki, Joanna Palma, Michał Stosik, Wiesław Deptuła

**Affiliations:** 1 Institute of Biology, University of Szczecin, 71-412 Szczecin, Poland; lukasz.baraniecki@phd.usz.edu.pl; 2 Doctoral School, University of Szczecin, 70-384 Szczecin, Poland; 3 Department of Biochemical Sciences, Pomeranian Medical University in Szczecin, 71-460 Szczecin, Poland; joanna.palma@pum.edu.pl; 4 Institute of Biological Science, Faculty of Biological Sciences, University of Zielona Góra, 65-516 Zielona Góra, Poland; m.stosik@outlook.com; 5 Institute of Veterinary Medicine, Faculty of Biological and Veterinary Sciences, Nicolaus Copernicus University in Toruń, 87-100 Toruń, Poland; wieslawdeptula@umk.pl

**Keywords:** platelets, platelet receptors, immune activation

## Abstract

The article characterises platelets, pointing out the role and contribution of their numerous receptors determining their specific and broad immune activity. Three types of platelet receptors are described, that is, extracellular and intracellular receptors—TLR (toll-like receptors), NLR (NOD-like receptor), and RLR (RIG-I-like receptor); extracellular receptors—selectins and integrins; and their other extracellular receptors—CLR (C-type lectin receptor), CD (cluster of differentiation), TNF (tumour necrosis factor), among others. Outlining the contribution of these numerous platelet receptors to the intravascular immunity, it has been shown that they are formed by their fusion with pathogen-associated molecular patterns (PAMPs), damage-associated molecular patterns (DAMPs), and lifestyle-associated molecular patterns (LAMPs). They are initiating and effector components of signal transduction of these cells, and their expression and quantity determine the specific and broad functions of platelets towards influencing vascular endothelial cells, but mainly PRRs (pattern recognition receptors) of blood immune cells. These facts make platelets the fundamental elements that shape not only intravascular homeostasis, as previously indicated, but they become the determinants of immunity in blood vessels. Describing the reactions of the characterised three groups of platelet receptors with PAMP, DAMP and LAMP molecules, the pathways and participation of platelets in the formation and construction of intravascular immune status, in physiological states, but mainly in pathological states, including bacterial and viral infections, are presented, making these cells essential elements in the health and disease of mammals, including humans.

## 1. Introduction

Platelets are cells that, because of their vulnerability at the site of injury, including infection, are the first effectors to form homeostatic barriers in the vasculature and are essential components of intravascular immunity [1,2,3,4,5,6,7,8,9,10,11]. These cells in mammals, including humans, are biconvex, nucleus-free morphotic elements of the blood, disc-shaped, surrounded by a cell membrane, and have a diameter of 2–5 µm, thickness of 0.5–1 µm, and surface area of 6–9 μm^2^ [6,12,13]. They arise from myeloid megakaryocytes and extramedullary megakaryocytes (lung, spleen), characterised by the former’s different transcriptomic, morphological, and functional properties [6,14]. The platelet membrane is composed of about 70% phospholipids, mainly phosphatidylcholine, phosphatidylserine, phosphatidylethanolamine, phosphatidylinositol, sphingomyelin, and cholesterol, as well as glycolipids and amorphous glycocalyx formed mainly by sialic acids, through which it acquires a negative charge [13,15,16,17,18]. In the cytoplasm of platelets, there is a unique arrangement of three types of microchannels, including those which are open to the surface of these cells and those which are not (i.e., dense (closed) and circular (peripheral), respectively), and five types of granules (i.e., α, δ, λ, peroxisomes, T), as well as two types of extracellular vesicles (EVs) (i.e., exosomes (microvesicles, MV, and microparticles, MP) and ectosomes), which are very richly equipped with a set of specific biologically active substances, in addition to typical organelles of mammalian cells, including, among others, mitochondria, lysosomes, Golgi apparatus, and ribosomes [5,13,14,15,17,18,19,20]. A feature peculiar to these cells is that their skeleton is characterised by such integrity that it does not change, even under the action of forces produced by blood flow [1,2,5,12,13,15,21,22,23]. This feature is conditioned by their microtubules and the microfilaments in their cytoskeleton, consisting of contractile proteins, mainly actin, spectrin, and myosin [13,15,17,18]. These structures (microtubules and microfilaments), together with microtubules at the periphery of these cells, maintain their disc-shaped shape under resting conditions, which, upon activation, changes, with the participation of coronins, to a spherical shape, with numerous pseudopodia (lamellipodia and filopodia), which is referred to as a spiny spherocyte [13,24]. Although platelets, as mentioned, do not have a cell nucleus, their genetic material in the form of 16 kb of mitochondrial DNA is present in 5–8 mitochondria and is rich in mRNA [14,22,25,26,27,28,29] and is also enriched with transcription factors, including the non-genomic transcription factor NFAT (nuclear factor of activated T-cells); hence, the mechanisms responsible for the course of transcription in these cells operate independently of the classical programs of this process [28,30,31]. Platelet activation, on the other hand, is conditioned by and related to their overabundant repertoire of specific extracellular and intracellular receptors (Table 1), through which they acquire a very high and broad activity, which is determined by biologically active substances contained in their five types of granules and two types of EVs [5,6,12,14,31,32]. The process of platelet activation itself occurs as a result of the interaction of their receptors with vascular endothelial cells, including the subendothelial layer, and the soluble substances released from their damaged cells, as well as PRRs (pathogen recognition receptors) of immune cells, pathogen-associated molecular patterns (PAMPs), damage-associated molecular patterns (DAMPs) and lifestyle-associated molecular patterns (LAMPs) molecules [5,14,18,33,34,35]. It should also be added that in the context of the platelet activation pathway, it is indicated and accepted that platelets’ critical feature and property is platelet–platelet interaction [32,36]. As a result of the interaction of platelets with the presented structures, there is an induction of their activity, which occurs due to an intracellular chain of reactions, mainly related to platelet NGP (neuronal guidance proteins), which makes them essential elements of intravascular homeostasis and immunity [2,3,4,6,7,8,9,10,11,12,13,14,17,18,19,21,29,31,32,33,36,37,38,39].

## 2. Platelet Receptors and Their Involvement in the Immune Activity of These Cells

**Figure 1:**
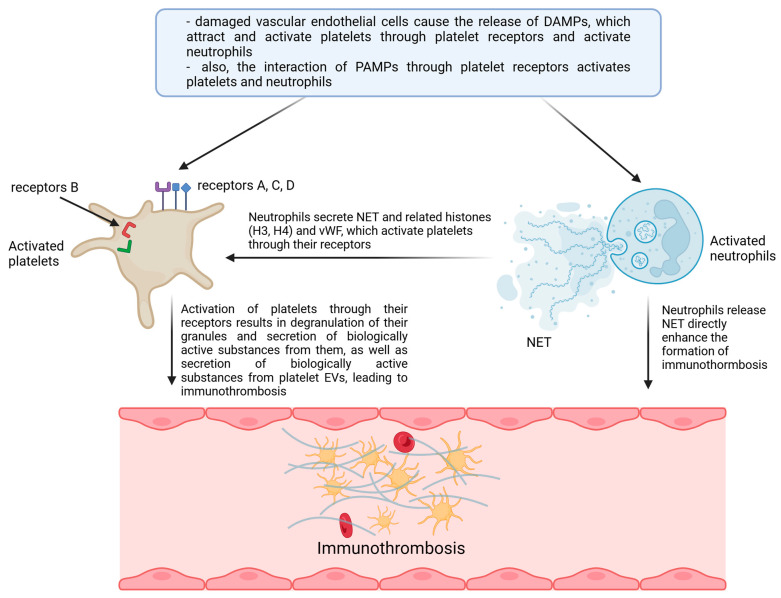
Involvement of platelets in the formation of immunothrombosis in blood vessels. **Abbreviations:** (**A**) extracellular receptors—TLR-1, 2, 4, and 6 (Tab. 1); (**B**) intracellular receptors—TLR-3, 7, 8, and 9, NLR, and RLR (Tab. 1); (**C**) extracellular selectin and integrin receptors (Table 1); (**D**) other extracellular receptors, e.g., CLR; DAMP—damage-associated molecular patterns; PAMP—pathogen-associated molecular patterns; vWF—von Willebrand factor, EV—extracellular vesicles. Created with BioRender.com.

**Figure 2:**
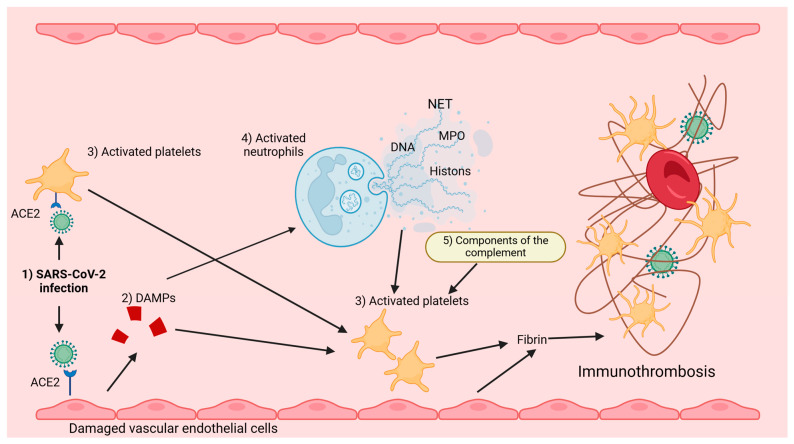
Some pathways of platelet involvement in the formation of immunothrombosis in blood vessels during SARS-CoV-2 infection. **Explanations:** (**1**) SARS-CoV-2 infecting platelets activates them, and infecting the vascular endothelium damages it, resulting in endothelial cells secreting DAMPs. (**2**) DAMPs attract activated platelets after SARS-CoV-2 infection to the damaged endothelial cells. (**3**) These activated platelets secrete polyphosphates and, together with vascular endothelial cells, promote fibrin formation and immunothrombosis. (**4**) In addition, neutrophils, activated by DAMPs during SARS-CoV-2 infection, secrete NETs, which interact with activated platelets toward the formation of immunothrombosis. (**5**) Furthermore, complement components (C3a, C5a) activated by SARS-CoV-2 virus infection, enhancing TF (tissue factor) expression, activate platelets for immunothrombosis. ACE2—Angiotensin-converting enzyme 2 receptor, MPO—Myeloperoxidase, NET—Neutrophil extracellular traps Created with BioRender.com.

Three types of receptors characterise platelets (Table 1), among which the first types are extracellular and intracellular TLRs (toll-like receptors) 1, 2, 3, 4, 6, 7, 8, and 9 and intracellular NLRs (nucleotide-binding oligomerisation domain-like receptors), including NODs (NOD-like receptors) 1 and 2, NLRC (NLR family CARD domain-containing proteins) 4 and NLRP (nucleotide-binding oligomerisation domain) 1–9, and RLR (RIG-I-like receptors), including RIG-I (retinoic acid-inducible gene-I-like receptors). In contrast, the second type of these receptors (Table 1) is extracellular selectin and integrin receptors, except that P-selectin (CD62P) receptors represent the former. At the same time, the latter are α2β1 glycoprotein receptors (GPIa/IIa—VLA2), αIIbβ3 (GPIIb/IIIa), the GPb-IX-V complex consisting of the glycoproteins GPIbα, GPIbβ, GPIX, and GPV, which form the human platelet antigen (HPA) receptor, and three other glycoprotein receptors, GPIβα, GPVI, and α6β1. Meanwhile, the third type of platelet receptors (Table 1) is formed by other extracellular receptors, which are represented by C-type lectin receptors (CLR), including CLEC-2 (C-type lectin-like type II transmembrane receptor) and DC-SIGN (dendritic cell-specific intercellular adhesion molecule-3 grabbing non-integrin), CD (cluster of differentiation) receptors, FcγRIIa (Fc immunoglobulin receptor), MHC (major histocompatibility complex) class I, GARP (glycoprotein A repetitions predominant), LPA (lysophosphatidic acid receptor) 1, 2, and 3, P2Y (purinoceptor) 1 and 12, PAR (protease-activated receptors) 1, 2, and 4, ADP (adenosine-diphosphate receptor), TxA (thromboxane), as well as receptors for IL (interleukins), that is, IL1β, IL1R1, and IL18Ra, cytokines such as TNF (tumour necrosis factor), IFNγ (interferon γ), and TGFBR (transforming growth factor beta receptor), and chemokines CCL—1, 3, and 4, CCL5, CXCL1, 2, 3, 4, 6, 7, 8, 12, 16, 17, 22, 24, and 26, ICAM (intercellular adhesion molecule) 1 and 2, JAM-A and C (junctional adhesion molecule A and C), PECAM (platelet endothelial cell adhesion molecule) 1, complement components (C) C1q, C8, C9, C3a, C3aR, and ACE2 (angiotensin-converting enzyme 2) receptors. These platelet receptors (Table 1) are the initiating and effector signal transduction components of these cells, the expression and abundance of which determines their specific and broad functions in the vascular environment towards vascular endothelial cells, blood cells, including immune cells, which makes them the fundamental elements shaping not only homeostasis but also intravascular immunity (Figure 1, Figure 2) [3,4,9,10,12,21,23,28,30,34,35,38,46,60,61,62,63].

### 2.1. Extracellular and Intracellular Platelet TLR, NLR, and RLR Receptors

These platelet receptors (TLRs, NLRs, and RLRs) and FcγRIIa and CLEC receptors representing other extracellular receptors of these cells (Table 1) are identified as immune markers. It has been shown that TLR, NLR, and RLR receptors, by binding to elements of the blood vessel environment and receptors that recognise the molecular patterns of pathogens (PRRs) of immune cells, as well as PAMP, DAMP, and LAMP molecules, condition, in blood vessels, anti-inflammatory and immune responses, including against germs [1,3,5,9,10,12,18,23,25,28,33,39,45,64,65,66,67] (Table 2). In humans, the extracellular platelet TLRs are TLR-1, 2, 4, and 6. In contrast, the intracellular receptors are TLR-3, 7, 8, and 9, found in more significant numbers in women than in men, and the intracellular NLRs represented by NOD 1, 2, NLRC-4, and NLRP 1–9 and the RLRs represented by RIG-I [11,25,28,30,38,64,65,68] (Table 2). Among extracellular platelet receptors, that is, TLR-1, 2, 4, and 6 (Table 1), TLR-4 receptors are relatively best known, which, especially in response to bacterial LPS, although also some viruses, cause in these cells, through the NF-κB factor, an intense synthesis of pro-inflammatory cytokines and expression of MHC class II antigens [3,5,6,14,18,23,69,70]. This process is aided by the participation of, among others, plasma-soluble CD14 receptors, as well as elements of the MyD88 (myeloid differentiation primary response 88) and TRIF (TIR-domain containing adapter-inducing interferon-β)-dependent pathway. The latter pathway stimulates platelets, increasing their surface P-selectin expression and releasing soluble CD40L (CD154) ligands and inflammatory mediators, including chemokines [18,69,70,71,72]. It has been shown that activation of platelets via the TLR-4 receptor, due to the interaction of Dengue virus PAMPs and DAMPs, including heat shock proteins (HSP) and HMGB1 (high-mobility group box 1), as well as ATP and NSP1 (non-structural protein 1), leads to platelet activation and results in the activation of the pyroptosis process [3,5,23,38,72]. On the other hand, when the TLR-2 receptors activates platelets due to the interaction of lipoprotein components of Gram-positive and Gram-negative bacteria and some viruses, these cells are stimulated in the synthesis of anti-infective substances [3,23]. It has been shown that, as a result of the interaction of Rickettsia africa and Porphyromonas gingivalis with platelets, through TLR-2 receptors, the release of soluble ligand CD40L (CD154) and chemokines CCL5 from platelets is induced, which activate platelets, mainly in terms of migration and chemotaxis [3,18,73]. In addition, it has been recorded that interaction through TLR-2 with PAMP of cytomegalovirus, Dengue, measles, and SARS-CoV-2 platelets have intense aggregation activation [3,18,23]. Such a state of platelets also arises after the combination of their TLR-2 receptors with elements of group B streptococci, leading to intense ADP release from them and their stimulation [74]. It is also indicated that the activation of platelets in terms of aggregation, adhesion, and chemokine secretion through unspecified TLR receptors arises from the interaction of cytomegalovirus PAMPs, the process of which also further activates the interaction of platelets with leukocytes, as a result of which the endocytosis of these viruses by leukocytes is intensified [3]. It has also been proven that the pathway of platelet activation through TLR receptors often leads to the formation of a heterodimer of the TLR-2 with TLR-1 and TLR-6, which forcefully activates them [71,75,76], a process that is also aided by their integrin receptors GPVI [38]. On the other hand, activation of platelets by TLR-2 and TLR-1 receptors, after their stimulation with unspecified substances of microbial origin, induces their prothrombotic response by increasing their adhesion capacity [23]. The analogous activation of platelets through the TLR-2 and TLR-6 receptors increases their activity in adhesion and aggregation [23]. It should be added that the stimulation of platelets, in terms of their aggregation, is associated with their transcription factor NFAT, which interacts with them through the thrombin receptor [23,28]. Meanwhile, the induction of platelet activity through TLR-2 and TLR-4 receptors, as a result of the interaction of LPS of Gram-negative bacteria and lipoteichoic acid (LTA) of Gram-positive bacteria, mainly enhances their aggregation and secretion and increases the expression of their activation receptor CD40 and CD62P (P-selectin) on platelets [3,18], which affects neutrophil activity in NETs and phagocytosis [5,9,18,20,25,26,65,68,77,78,79,80].

Regarding intracellular TLRs represented by TLR-3, 7, 8, and 9 (Table 1), platelet activation, as with previous TLRs, also varies. It has been shown that the activation of these cells occurs via TLR-3 reacting with dsRNA viral nucleic acid ligands, TLR-7 reacting with ssRNA viral nucleic acid ligands, and TLR-8 reacting with ssRNA viral nucleic acid ligands and bacterial RNA and chitin. In the case of TLR-9 receptors, platelet activation occurs after binding to DNA viral ligands, mainly unmethylated DNA [7,9,18,23,25,38,64,68,81,82]. These studies have also shown that stimulation of platelets via TLR-3, poly (I:C) (synthetic double-stranded RNA), leads to increased platelet aggregation and increased synthesis of TNFR1 (tumor necrosis factor receptor 1), as well as activation of NK-κB signaling [23,83]. On the other hand, stimulation of these cells via TLR-7 with ssRNA viruses enhances adhesion. It increases the expression of their surface selectins and the secretion of substances from their α-granules, enhancing the inflammatory process by increasing the combative activity of PMN cells and their NETs [7,18,23,38,82]. Meanwhile, activation of platelets via TLR-8 with ssRNA viruses stimulates TNFR1 synthesis in them [23], while their stimulation via TLR-9 with DNA viruses, bacterial LPS, or DAMP leads to increased expression of their receptors CD62P (P-selectin) and CD63 and integrin markers αIIbβ3 [18]. In addition, it has been recorded that viral or bacterial “material” interacting with platelets, through their TLR-3, 7, 8, and 9, leads to increased secretion of substances from their α, dense, and T granules, which leads to increased innate and acquired immunity in blood vessels [14,18,38,84]. It has also been recorded that platelet activation by viruses and intracellular bacteria [2] affects thrombin, resulting in increased secretion of TLR-9 receptors from their T cell granules [85].

On the other hand, in the case of intracellular NLR receptors of platelets (Table 1), it was registered that their stimulation through the NOD2 receptor, muramyl dipeptide (MDP) of *Gram-positive* and *Gram-negative* bacteria, causes the release of ATP from them and increases their aggregation and clot formation, which is a defense of the macroorganism in terms of mechanical “binding” of bacterial and viral antigens [5,23,59,78]. Furthermore, stimulation of platelets, through the intracellular receptor NLRP3, the main element of inflammasome activation, with PAMP of RNA viruses, including Dengue virus, triggers a “state” in blood vessels leading towards “controlled” inflammation, causing activation of caspase-1, resulting in cleavage of pro-IL-1β and pro-IL-18 and formation of active forms of IL-1β and IL-18 [33,40,59,86]. It is also indicated that intracellular platelet NLRP3 and NOD2 receptors (Table 1) and IL-1β may also be involved in prothrombotic processes [38]. Meanwhile, activation of platelets via intracellular RLR receptors, including RIG-I (Table 1), due to PAMP interaction of ssRNA and dsRNA viruses, results in strong induction of antiviral activity of these cells [3,66].

### 2.2. Platelet Extracellular Selectin and Integrin Receptors

Among these platelet receptors (Table 1), selectin receptors are represented by receptors for P-selectin (CD62P), which show specificity to subendothelial vascular elements, that is, fibronectin, P-selectin glycoprotein ligand-1 (PSGL-1) molecules CD15, CD11/18, and CD34, glycosylation-dependent cell adhesion molecule-1 (GlyCAM-1), MadCAM-1 (mucosal vascular addressin cell adhesion molecule 1), complement component C3b, as well as Th1 cell receptors, DCs, PMNs, and monocytes. As a result of these reactions, platelet activity is increased, particularly platelet aggregation and adhesion [13,30,34,51,68,78,80,87,88,89,90,91,92] (Table 2). It has been shown that the reaction of platelets, via the P-selectin receptor (CD62P) with vascular endothelial cells, during viral infection causes an increase of up to 10,000 of these molecules on the platelet, leading to an increase in the adhesion capacity of platelets to monocytes and T lymphocytes [9,13,67,87]. In addition, during viral infection, due to conformational changes within the integrin receptor αIIbβ3 (GPIIb/IIIa) of platelets, expression of the receptor genes for P-selectin (CD62P) is induced, resulting in increased activity of these cells [30,61,93] (Table 2).

However, among platelet integrin receptors (Table 1), it has been shown that their α2β1 (GPIa/IIa—VLA2) receptor, referred to as a homeostatic receptor, when combined with their specific ligands, such as collagen or laminin, enhances activation of vascular endothelial cells and blood leukocytes [25]. On the other hand, when the receptor α2β1 (GPIa/IIa—VLA2) binds to rotavirus PAMPs, there is strong platelet activation, which is further supported by their glycoprotein receptor GPVI [48]. In addition, it has been shown that binding of platelets via the characterised α2β1 receptor (GPIa/IIa—VLA2) to thrombospondin and fibrinogen activates them, although this reaction is also assisted by the integrin receptor GPIβα of these cells [6,25,94]. It was discussed that platelet receptor α2β1 (GPIa/IIa—VLA2), binding to vitronectin during bacterial and viral infections, induces platelet activity very intensively [4,21,30,34,42,48,60]. When integrin receptor αIIbβ3 (GPIIb/IIIa) platelets (Table 1) fuse with a specific receptor such as fibrinogen, during infections with *Staphylococcus (Staph.) aureus*, *Staph. epidermidis*, *Staph. pseudintermedius*, *Streptococcus (S) agalactiae*, *S. gordonii, S. mutant*, *S. pneumoniae*, *S. pyogenes*, and *S. sanguinis*, as well as *Borrelia burgdorferi*, *Chlamydia pneumoniae*, *Mycobacterium tuberculosis*, and *Candida albicans* or infections with SARS-CoV-2 virus, hantavirus, and adenoviruses, this also results in increased activity of these cells [3,48,95]. On the other hand, platelets, through this receptor with collagen, activate vascular endothelial cells and blood leukocytes, which affects homeostasis in blood vessels [25,55,94]. It has been shown that the αIIbβ3 (GPIIb/GPIIIa) platelet receptor, together with the integrin receptor GPIbα and the FcγRIIa receptor (Table 1), when combined with the soluble ligands of these cells, form the most efficient “pathways” to induce platelet activity during bacterial and viral infections [3,5,41,95]. It has also been proven that the integrin receptor αIIbβ3 (GPIIb/GPIIIa), together with the platelet integrin receptor α6β1 [19,34], as a result of increased fibrinogen, induces platelet aggregation and adhesion [3,9,13,19,21,26,30,34,42,43,51,61,96,97]. In contrast, the association of the receptor αIIbβ3 (GPIIb/IIIa) with MMP-2 (matrix metalloproteinases-2) of blood leukocytes leads to their activation in a pro-inflammatory direction [13,30,60], as does its association with low-density lipoprotein (LDL) [4,9,13,19,30,93,98]. It is also indicated that the most important platelet receptor, in addition to the vWF (von Willebrand factor) receptor, is the platelet integrin receptor GPIβα (Table 1), which determines the maintenance of intravascular homeostasis [3,48,99,100], which is also largely influenced by thrombin, coagulation factors XII and XI, and kininogen [2,101]. Meanwhile, the platelet integrin receptor in the form of the GPIb-IX-V complex, consisting of four glycoproteins (GPIbα, GPIbβ, GPIX, and GPV) that constitute the HPA receptor of these cells (Table 1), is abundantly represented on their surface (about 30,000 copies in the form of GPIbβ and GPIX glycoprotein and 15,000 copies in the form of GPV glycoprotein) and, as a result of binding to the PAMP of germs, activates platelet adhesion, as recorded in patients affected by systemic lupus erythematosus (SLE) [6,34]. It has also been shown that the glycoproteins GPIbα and GPIbβ of the GPIb-IX-V complex, a platelet HPA receptor (Table 1), are specific receptors that activate these cells during infection with *S. sanguinis*, *S. gordoni*, and *S. oralis* [3]. It has also been registered that the combination of GPIbα glycoprotein with fibrinogen, fibronectin, and vWF factor prominently increases, with the participation of macrophage-1 antigen (macrophage-1 marker), the aggregation of platelets with neutrophils, resulting in increased intravascular immunity [18,30,44]. It has also been recorded that deficiency of platelet GPIbα glycoprotein in mouse sepsis can result in decreased platelet–neutrophil and platelet–monocyte interactions, which reduces the synthesis of TNF-α, IL-6, and the chemokines MCP-1 (monocyte chemoattractant protein-1) (CCL-2) and MIP-1β (macrophage inflammatory protein-1β) (CCL-4), as well as other inflammatory factors [15,17,43,87,102]. On the other hand, dysfunction or non-existence of one of the three glycoproteins, that is, GPIbα, GPIbβ, and GPIX, among the four that comprise the GPIb-IX-V complex, a platelet HPA receptor, along with deficiency of integrin receptor αIIbβ3 (GPIIb/IIIa), also an HPA receptor element (Table 1), is the cause of Bernard–Soulier syndrome (BSS), which is manifested by spontaneous and post-traumatic dermal-mucosal haemorrhagic blemishes, recorded in von Willebrand disease [19,34,42,44]. Furthermore, it has been proven that the glycoprotein GPV component of the GPIb-IX-V complex (Table 1), by affecting thrombin synthesis and “production” of polymerised fibrin, influences the process of blood clotting and clot formation, which significantly enhances intravascular immunity [11]. It is assumed that for platelet activation to occur through integrin receptors that form the HPA receptor (Table 1), they must interact with these cells’ FcγRIIa, TLR, and CLEC2 receptors [3,55]. It should be added that the receptors forming the platelet HPA receptor are strongly generated after exposure to foreign platelets in the production of alloantibodies [103]. In addition, the platelet HPA receptor, especially under conditions of vWF receptor-dependent arterial stenosis and receptor-dependent venous stenosis for collagen and fibrinogen, affects the adhesion of these cells, which is critical in areas of vasoconstriction [3,11,44]. It is assumed that due to vascular constriction and contraction, fibronectin released from platelet α granules deposited on blood vessel walls further mediates the so-called “protein wave” of homeostasis in vessels [11]. Moreover, it has been recorded that platelet GPVI integrin receptors (Table 1), combining with collagen and thrombospondin, increase the aggregation of these cells, a state that is mediated by the action of immunoreceptor tyrosine-based activation motif (ITAM) [13,21,34,42,47,66,87,103]. It has been shown that during platelet activation through this GPVI receptor, these cells release ADP and TxA2, which strongly activate blood leukocytes and increase intravascular immunity status. The combination of the platelet GPVI receptor together with the integrin receptor α2β1 (GPIa/IIa—VLA-2) and the ICAM-1 and PECAM-1 receptors (Table 1) of these cells with fibrinogen, fibronectin, and vitronectin leads to the formation of “cross bridges” that greatly enhance platelet activity [4,13,21,26,30,34,42,55,60,96,97]. Furthermore, platelet activation through this receptor, GPVI, has been recorded during SFTS (severe fever with thrombocytophenia syndrome) and Klebsiella pneumoniae infection [3].

### 2.3. Other Extracellular Platelet Receptors

Among these platelet receptors (Table 1) are C-type lectin-like receptors, also referred to as CLR-type lectin-like receptors, e.g., CLEC-2 (C-type lectin-like receptor 2) and DC-SIGN (dendritic cell-specific intercellular adhesion molecule-3 grabbing non-integrin) receptor (Table 1), which bind to the PAMPs of various viruses and cause strong platelet activation [3,24,46,56] (Table 2). It has been shown that the fusion of platelets, through CLEC-2- and DC-SIGN-type receptors with PAMPs of Dengue, Ebola, Marburg, influenza (H1N1, N5N1), SARS-CoV, SARS-CoV2, and HIV viruses, causes intense secretion of biologically active substances from their α and dense granules and EVs [3,14,23,48,57,58]. Moreover, the association of platelets, through their unspecified CLR-type receptors, with DAMP molecules leads to increased expression of pro-inflammatory cytokines of these cells and leukocytes and an increase in so-called “sterile” inflammation [4,51,64,91]. It should also be added that the platelet CLR-type lectin receptor CLEC2, during embryonic development activated by podoplanin, affects the separation of blood and lymphatic vessels, while during individual development, it participates in clot formation [23,48]. It has also been proven that blocking the lectin receptor CLR-DC-SIGN and the platelet receptor FcγRIIa can inhibit their activation during infection, which can be used in disease states characterised by excessive activity of these cells [28].

Among other extracellular platelet receptors (Table 1), the receptors CD116 and CD18 have been shown to impact the interaction of these cells with leukocytes, resulting in an increased inflammatory reaction [34,51] (Table 2). In contrast, the CD40 receptor of these cells (Table 1), which binds to DCs and B lymphocytes, plays a crucial role in switching antibody isotypes on B lymphocytes from IgM to IgG, mainly IgG1, IgG2, and IgG3, although also IgA, and influences increased activity of TCD8^+^ lymphocytes and platelets themselves [6,8,11,33,45,91] (Table 2). The platelet CD40 receptor, during adenoviral infection, has been shown to cause changes in B lymphocytes, mainly in immunoglobulin class G isotopes and A [6,26,104,105], and its expression is conditioned by the MHC class I receptor and integrin receptor αIIbβ3 (GPIIb/GPIIIa), P-selectin, coronins, MMP (matrix metalloproteinase) family protein, and actin polymerisation [6,24,106]. In addition, it has been recorded that during adenoviral infection, the expression of ICAM1, VCAM1, and CCL2 receptors on platelets is also increased, leading to an enhanced inflammatory response of vascular endothelial cells [8,11,33]. This condition is also associated with increased expression of ICAM1, VCAM1, and CCL1 receptors on these cells, leading to exposure of the vascular endothelium and release of inflammatory mediators including IL-2, IL-8, CCL-2, and selectin E and P [4,33].

In the case of FcγRIIa receptors also belonging to other extracellular platelet receptors (Table 1), which are referred to as immune receptors, it has been shown that by binding to the Fc fragment of immunoglobulin class G, A, and E, they increase during the phagocytosis process of these cells the opsonisation of bacteria *Staphylococcus aureus* and *Helicobacter pylori*, among others, a process that is also promoted by the integrin receptor αIIbβ3 (GPIIb/IIIa) of these cells, as well as MMP family proteins [3,5,44,48,64,96,107] (Table 2). Such “binding” of platelets via the FcγRIIa receptor to bacterial antigens also leads to strong activation of complement system proteins, an essential element in anti-infective immunity, including bacterial immunity [6,23,91]. The platelet FcγRIIa receptor has been shown to enhance the migration and recruitment of these cells when combined using IgG with PAMPs of microorganisms, including *S. bovis*, *S. equinus*, *S. sanguinis*, and *Bacillus athracis*, together with the integrin receptor αIIbβ3 (GPIIb/GPIIIa) [3,12,39,41]. Its combination with the influenza virus (H1N1) and SARS-CoV-2 virus may also lead to adverse effects, as such a condition increases platelet adhesion to the endothelium and enhances blood coagulation, which may lead to systemic shock [3,108].

Also belonging to other extracellular platelet receptors, MHC class I receptors (Table 1) affect the course of infection in *Plasmodium* sp. because they enhance the presentation of these protozoan antigens to CD8+ T lymphocytes [33,109,110]. MHC class I receptors can also present, but only with CD86 molecules and other antigens, to CD8+ T lymphocytes and activate platelets [6] (Table 2). Moreover, platelet GARP (glycoprotein A repetitions predominant) receptors (Table 1) belonging to the discussed receptors, through Treg lymphocytes and TGFβ (transforming growth factor β) cytokine, activate platelets and blood immune cells [4,9,19,30,111] (Table 2). Similar to GARP receptors, lysophosphatidic acid receptors (LPA_1_, LPA_2_, and LPA_3_) (Table 1), by binding to lysophosphatidic acid found in oxidised LDL (mox-LDL—low-density lipoproteins), intensively activate platelets (Table 2), as recorded in patients with advanced atherosclerotic lesions [47]. It has been shown that purinergic receptors, P2Y_1_ and P2Y_12_ platelets (Table 1), after binding to the ADP receptors of these cells, in which the non-genomic transcription factor NFAT is also involved [28], cause increased platelet aggregation and stimulate immune cells and, together with coronins, alter the shape and size of platelets, thereby enhancing their defensive functions [8,19,24,34,59]. In the case of the receptors in this group, that is, PAR 1, 3, and 4 of platelets (Table 1), it has been shown that their combination with thrombin causes, mainly through the most dominant receptor among them, PAR1 [112], found on these cells in about 2500 copies, activation of platelets [13,15,19,49,113]. In addition, the PAR1 and PAR4 receptors, together with P2Y_1_ and P2Y_12_, as well as ADP and the receptor for platelet thromboxane (TxA) (Table 1), greatly increase their aggregation and affect conformational changes mainly in their integrin receptors, as well as in their pseudopodia (lamellipodia and filopodia), which increases the defensive properties of platelets [4,6,8,9,12,30,34,42,46,47,111] (Table 2). It has also been recorded [12] that during bacterial sepsis, vascular permeability is increased via TxA receptors from this group of platelet receptors (Table 2), which can result in acute damage in internal organs, including the lungs [12]. On the other hand, among the discussed other extracellular platelet receptors, which are receptors for interleukin IL1β, IL1R1, and IL18Ra, as well as cytokines TNF (tumour necrosis factor), IFNγ, TGFβR (transforming growth factor beta receptor), and chemokines (CCL-1, 3, 4, CCL5 and CXCL1, 2, 3, 4, 6, 7, 8, 12, 16, 17, 22, 24, and 26) (Table 1), as a result of their combination with “foreign substances” formed by acute inflammation or infections, mainly viral, there is strong platelet activity [4,5,9,30,33,45,91,114] (Table 2). It has been recorded that the abovementioned group of platelet cytokine and chemokine receptors, as a result of binding to viral and bacterial PAMPs, cause increased platelet activity, including their interaction with phagocytic cells, resulting in, among other things, a greater engulfing and cidal capacity of these cells, leading to an increased status of intravascular immunity [5,12,33,91]. In the case of receptors in this group, platelet IL1R1 (Table 2) has been registered as having a combination with bacterial LPS that increases platelet aggregation and adhesion [84,94]. It has also been proven that platelet cytokine receptors, activating Treg lymphocytes, strongly induce the synthesis of IFNα, IFNβ, IL1β, IL10, and IL4, thereby enhancing intravascular immunity [5,33,38]. On the other hand, platelets binding through chemokine receptors such as CXCL4 increase their activity, including the recruitment of PMN cells, monocytes, and T lymphocytes to the site of inflammation [12,33]. It should be added that the chemokine CXCL4 is a factor that can reduce inflammation because if it is derived from young mice (3 mo old) and given to adults (20 mo old), it has a rejuvenating effect on them, as it reduces the amount of pro-inflammatory factors (TNF, C1q) as well as markers of microglia activity in the hippocampus [115]. Furthermore, the combination of ICAM-1 and -2, JAM-A and C and platelet PECAM-1 receptors, which are also among the receptors discussed (Table 1), with “foreign substances” increases the aggregation and adhesion of these cells [34,42,68] (Table 2). Similarly, the receptors for complement components—C1q, C8 and C9, C3a and C3aR (Table 1)—belonging to these platelet receptors, when combined with, for example, *S. sanguinis*, *Staph. aureus*, or SARS-CoV-2 virus, causes their strong activation, mainly in terms of aggregation [3,116,117] (Table 2). It has been recorded that the receptor for complement components C3a and C3aR, influencing platelet adhesion, affects blood coagulation and stimulates endothelial cells in an anti-angiogenic direction [116,117]. It has been recorded that in the case of platelet receptors for C1q complement components, the stimulation of these cells occurs through the classical pathway of complement action, while in the case of complement components C8 and C9, the formation of the C5b-C9 complex, the so-called MAC (membrane attack complex), which causes the formation of trans-membrane channels, “builds” platelets into the mechanism of their destructive action against microorganisms [5,12]. In addition, it is indicated that the MAC complex also stimulates the blood vessel environment towards the release of TF and vWF, which are elements of blood coagulation [12]. However, they also affect the release of substances from EVs of platelets [14,23]. Regarding ACE2 receptors, within other platelet surface receptors (Table 1), this receptor has been found to bind to the SARS-CoV-2 virus protrusion protein, allowing the virus to enter platelets, leading to induction of the activity of these cells, mainly in terms of natural immunity [3,5,118] (Table 2).

## 3. Summary

Platelets are cells that, although lacking a cell nucleus, are equipped with typical mammalian cell structures, including being remarkably enriched in extracellular and intracellular receptors (Table 1). These receptors, as a result of their interaction with vascular endothelial cells and immune cells in the blood, as well as molecules associated with PAMP, DAMP, and LAMP, shape and condition not only intravascular homeostasis but also immunity, including anti-infective, autoimmune, anti-allergic, and antitumour, making them essential elements in health and disease in mammals, including humans.

## Figures and Tables

**Table 1:** Platelet receptors [1,2,3,5,8,9,12,13,18,19,23,30,38,40,41,42,43,44,45,46,47,48,49,50,51,52,53,54,55,56,57,58,59].

Lp.	Extracellular and intracellular TLR, NLR, and RLR receptors	(a) Extracellular receptors: TLR-1, 2, 4, 6 (b) Intracellular receptors: TLR-3, 7, 8, 9, NLR in this NOD1, NOD2, NLRC-4, and NLRP 1–9 and RLR in this RIG-I
1.		
2.	Extracellular selectin and integrin receptors	(a) Selectin markers: markers for P-selectin (CD62P) (b) Integrin markers: α2β1 (GPIa/IIa—VLA2), αIIbβ3 (GPIIb/IIIa), complex GPb-IX-V; it is GPIbα, GPIbβ, GPIX, GPV which form the HPA receptor and receptors GPIβα, GPVI, α6β1
3.	Other extracellular receptors	Lectin receptor type C; it is the CLR in this CLEC-2 and DC SIGN, and also CD116, CD18, CD40, FcγRIIa, MHC class I, GARP, LPA_1,_ LPA_2,_ LPA_3_, P2Y_1,_ P2Y_12_, PAR-1,3,4, ADP, TxA, IL1β, IL1R1, IL18Ra, TNF, IFNγ, TGFβR, CCL-1, 3, 4, 5, CXCL-1, 2, 3, 4, 6, 7, 8, 12, 16, 17, 22, 24, 26, ICAM-1, ICAM-2, JAM-A, JAM-C, PECAM1, C1q, C8, C9, C3a, C3aR, and ACE-2

**Abbreviations:** TLR—tool-like receptor, NOD—nucleotide-binding and oligomerization domain, NLR—NOD-like receptors, NLRC—NLR family CARD domain-containing protein, NLRP—nucleotide-binding oligomerization domain leucine-rich repeat and pyrin domain containing, RLR—RIG-I-like receptors, RIG-I—retinoic acid-inducible gene-I-like receptors, CD—cluster of differentiation, HPA—human platelet antigen, C-CLR—C-type lectin receptor, CLEC-2—C-type lectin-like type II transmembrane receptor, DC-SIGN—dendritic cell-specific intercellular adhesion molecule-3 grabbing non-integrin, FcγFRIIa—receptor Fc immunoglobulin, MHC—major histocompatibility complex, GARP—glycoprotein-A repetitions predominant, LPA—lysophosphatidic acid, receptor P2Y—purinergic receptors, PAR—protease-activated receptors, ADP—adenosine-diphosphate receptor, TxA—thromboxane receptor A, IL- interleukin, TNF—tumor necrosis factor receptor superfamily, IFNγ—interferon gamma, TGFβR—transforming human growth factor beta receptor, CCL—chemokine (CCL-1, 3, 4, CCL5, CXCL-1, 2, 3, 4, 6, 7, 8, 12, 16, 17, 22, 24, 26), ICAM—intercellular adhesion molecule, JAM-A—junctional adhesion molecule A, JAM-C—junctional adhesion molecule C, PECAM—platelet endothelial cell adhesion molecule, C—complement, ACE-2—angiotensin-converting enzyme 2.

**Table 2:** Contribution and role of platelet receptors in the immune function of these cells.

Platelet Receptors		Pathways of Influence on Platelets	Action Effect	Involvement in Pathological States/Diseases
Extracellular and intracellular	TLR-1, 2, 4, 6	Increase the synthesis of pro-inflammatory cytokines, MHC class II markers, CD40, P-selectin, ligand for CD40L, CCL5, and aggregation, adhesion, migration, and chemotaxis, through the NF-κB factor, Myd88, and TRIF pathway. Also enhance phagocytosis and NET of platelets and endocytosis of viruses by these cells and leukocytes.	Enhance antibacterial and antiviral immunity, non-infectious and metabolic diseases	G+ and G- bacterial and viral infections, e.g., Dengue virus, and reaction to DAMP and LAMP patterns.
	TLR-3,7,8,9	Enhance aggregation, platelet adhesion, and the expression of TNFR1, selectin, and CD receptors on these cells, through factor NF-κB. Stimulate secretion of substances secreted from platelet dense granules and T cells, and increase the cidal capacity and NET of PMN cells.	Enhance antiviral and antimicrobial immunity, mainly against intracellular bacteria and non-infectious and metabolic diseases	Infections with ssRNA, dsRNA, DNA viruses, and intracellular bacteria and reaction to DAMP and LAMP patterns
	NLR	They enhance platelet aggregation and clot formation and stimulate the synthesis of IL-1β and IL-18 and the formation of “controlled” inflammation, with the help of the NOD2 receptor and NLRP3.	Enhance antibacterial and antiviral immunity and non-infectious and metabolic diseases	Infections with intracellular bacteria and viruses, mainly RNA viruses, and reaction to DAMP and LAMP patterns
	RLR	Stimulate antiviral immune mechanisms.	Enhance anti-infective immunity and non-infectious and metabolic diseases	Infections with ssRNA and dsRNA viruses and reaction to DAMP and LAMP patterns
Extracellular	Selectin	Activate vascular endothelial elements including fibrinogen, PSGL-1 ligand, and CD receptors and enhance Th1, DC, PMN, and MN cell activity, as well as platelet aggregation and adhesion.	Optimize intravascular homeostasis and enhance antiviral immunity in infections and vascular diseases	Viral infections
	Integrin	Enhance the activity of vascular endothelial cells and blood leukocytes through, among others, ITAM receptors and affect platelet aggregation and adhesion, as well as their secretion of ADP and TxA2. Enhance the activity of vascular endothelial cells and blood leukocytes through, among others, ITAM receptors and affect platelet aggregation and adhesion, as well as their secretion of ADP and TxA2.	Enhance antibacterial and antiviral immunity and stabilize intravascular homeostasis, vascular diseases, and autoimmune diseases	Systemic lupus erythematosus (SLE), Bernard–Soulier syndrome (BSS), SFTS syndrome, autoantibody production
Other extracellular	Lectin receptor type C	Increase the synthesis of pro-inflammatory cytokines by platelets and leukocytes, as well as increase the secretion of substances from α-granules, dense, and EV platelets	Enhance anti-infective immunity mainly against viruses in infections and non-infectious and metabolic diseases	Dengue virus infections, Ebola, Marburg, influenza, SARS-CoV-2, HIV, and reaction to DAMP patterns
	CD receptors	They affect isotype switching from IgM to IgG and IgA, increase TCD8 and platelet activity and vascular endothelial cell secreting, as well as IL-2, IL-8, CCL-2, and selectin G and P.	Enhance anti-infective immunity mainly against viruses in infections and vascular diseases	Adenovirus infections
	FcγRIIa, MHC class I, GARP, and lysophosphatidic acid (LPA_1_, LPA_2_, LPA_3_)	Activate platelets and Treg cells and enhance TGFβ secretion through, among others, the CD86 receptor.	Enhance anti-infective immunity in infections and parasitic infections and vascular diseases	Bacterial infections, influenza virus, SARS-CoV-2 infections, and *Plasmodium *sp. infections and atherosclerotic lesions
	P2Y_1_, P2Y_12_ receptors	They activate platelet aggregation via NFAT and stimulate immune cells and cause platelet shape changes with the contribution of coronins.	Enhance anti-bacterial immunity in bacterial infections	G- bacterial infections
	PAR 1, 3, 4, TxA receptors	Enhance the defensive attributes of platelets, cause a change in vascular permeability and a conformational change in integrin receptors and platelet pseudopodia.	Enhance anti-bacterial immunity in bacterial infections and vascular diseases	Bacterial infections
	Receptors for IL-1β, IL-1R1, IL-18α, TNF, INFγ, TGFβR, CCL-1,3,4,5, CXCL-1,2,3,4,6,7,8,12, 17,22,24,26, ICAM1 i 2, JAM-A i C, PECAM1, C i ACE-2	Activate aggregation, adhesion, engulfment and cidal capacity and secretion of platelet EV substances and increase TF, vWF release, and blood coagulation. Increase leukocyte chemotaxis, activate Treg toward synthesis of, among others, TNF-α, and activate blood vessel cells toward angiogenic.	Enhance antiviral and anti-bacterial immunity—mainly natural in infections and vascular diseases	Viral and bacterial infections

**Abbreviations:** TLRs—toll-like receptors, NLR—NOD-like receptor, RLR—RIG-I-like receptor, CD—cluster of differentiation, FcγRIIa—Fc immunoglobulin receptor, MHC—major histocompatibility complex, GARP—glycoprotein A repetitions predominant, LPA—lysophosphatidic acid receptor, P2Y—purinergic receptors, PAR—protease-activated receptors, TxA—thromboxane, IL—interleukin, TNF—tumor necrosis factor, INF—interferon, TGFβR—transforming human growth factor beta receptor, CCL—chemokine, CXCL—chemokines in the CXC subfamily, ICAM—intercellular adhesion molecule, JAM—junctional adhesion molecule, PECAM—platelet endothelial cell adhesion molecule, C—complement, ACE-2—angiotensin-converting enzyme 2, NET—neutrophil extracellular traps, TNFR1—tumor necrosis factor 1, NOD—nucleotide oligomerization domain receptors, NLRP—nucleotide-binding oligomerization domain, PSGL-1—P-selectin glycoprotein ligand-1, Th—T helper cell, DC—dendretic cells, PMN—polymorphonuclear cells, MN—mononuclear cells, ITAM—immunoreceptor tyrosine-based activation motif, ADP—adenosine diphosphate, EV—extracellular vesicles, Ig—immunoglobulin, TCD8—CD8 T cells, Treg—regulatory T cells, TGFβ—transforming growth factor β, NFAT—nuclear factor of activated T-cells, TF—tissue factor, vWF—von Willebrand factor, DAMP—damage-associated molecular patterns, LAMP—lifestyle-associated molecular patterns, G+—Gram-positive, G-—Gram-negative.
